# Recreational physical activity and breast cancer risk by menopausal status and tumor hormone receptor status: results from the Nurses’ Health Studies

**DOI:** 10.1007/s10549-023-07238-x

**Published:** 2024-04-09

**Authors:** Renée T. Fortner, Kristen D. Brantley, Shelley S. Tworoger, Rulla M. Tamimi, Bernard Rosner, Michelle D. Holmes, Walter C. Willett, A. Heather Eliassen

**Affiliations:** 1 Department of Research, Cancer Registry of Norway, Norwegian Institute of Public Health, Majorstuen, Postbox 5313, 0304 Oslo, Norway; 2 Division of Cancer Epidemiology, German Cancer Research Center, Heidelberg, Germany; 3 Channing Division of Network Medicine, Brigham and Women’s Hospital and Harvard Medical School, Boston, MA USA; 4 Department of Epidemiology, Harvard T.H. Chan School of Public Health, Boston, MA USA; 5 Department of Cancer Epidemiology, Moffitt Cancer Center, Tampa, FL USA; 6 Department of Population Health Sciences, Weill Cornell Medicine, New York, NY USA; 7 Department of Biostatistics, Harvard T.H. Chan School of Public Health, Boston, MA USA; 8 Department of Nutrition, Harvard T.H. Chan School of Public Health, Boston, MA USA

**Keywords:** Breast cancer, Risk, Physical activity, Menopausal status, Hormone receptor status

## Abstract

**Purpose:**

Physical activity is associated with lower breast cancer risk, especially in postmenopausal women. Associations in premenopausal women are less well established.

**Methods:**

We evaluated recreational physical activity and breast cancer risk in the Nurses’ Health Study (NHS) and NHSII (187,278 women; n = 12,785 breast cancers; follow-up: NHS = 1986–2016, NHSII = 1989–2017) by menopausal status and estrogen (ER) and progesterone (PR) receptor status. Physical activity was evaluated as updated cumulative average of metabolic equivalent of task (MET)-h/week. Cox proportional hazards models were used to estimate multivariable hazard ratios (HR) and 95% confidence intervals (CI).

**Results:**

Recreational physical activity was inversely associated with breast cancer risk in pre- and postmenopausal women. Higher activity levels were associated with lower risk of ER+/PR + breast cancer in both pre- and postmenopausal women (e.g., total recreational activity, ≥ 27 vs < 3 MET-h/week, premenopausal HR = 0.83, 95%CI = (0.70–0.99), postmenopausal HR = 0.86 (0.78–0.95); p_heterogeneity_ = 0.97). Results were attenuated with adjustment for current body mass index (BMI) among postmenopausal, but not premenopausal, women (e.g., ≥ 27 vs < 3 MET-h/week, premenopausal HR = 0.83 (0.69–0.98); postmenopausal HR = 0.95 (0.85–1.05); p_heterogeneity_ = 0.99). In analyses of moderate-vigorous activity and breast cancer risk, no heterogeneity by menopausal status was observed (p_het_ ≥ 0.53; e.g., ≥ 27 vs < 3 MET-h/week, ER+/PR+, premenopausal HR = 0.88 (0.69–1.11); postmenopausal HR = 0.71 (0.58–0.88). No associations were observed for ER−/PR− disease.

**Conclusions:**

Recreational physical activity was associated with lower breast cancer risk in both pre- and postmenopausal women, supporting recreational physical activity as an accessible, modifiable exposure associated with reduced breast cancer risk regardless of menopausal status.

**Supplementary Information:**

The online version contains supplementary material available at 10.1007/s10549-023-07238-x.

## Introduction

Higher levels of physical activity are consistently associated with lower risk of breast cancer for postmenopausal women, while associations for premenopausal women remain less clear [1], in large part due to the relatively small number of premenopausal cases with pre-diagnosis activity data in individual prospective cohort studies. The World Cancer Research Fund International (WCRF): Continuous Update Project (CUP) 2018 Expert Report [2] summarized associations between physical activity and breast cancer and reported inverse associations between vigorous activity and both pre- and postmenopausal breast cancer, while total and recreational activity were only significantly associated with postmenopausal disease and with the associations for premenopausal women classified as “limited-suggestive” [1, 2]. In contrast, a more recent study in the UK Biobank reported a 17–23% lower breast cancer risk with higher levels of activity in pre- and postmenopausal women [3]. Inverse associations for pre-/perimenopausal [4] and postmenopausal women are further supported by results from Mendelian randomization studies [5].

Beyond the somewhat conflicting results by menopausal status for breast cancer overall, few studies have evaluated physical activity and breast cancer risk by hormone receptor status in premenopausal women [6]. Differing associations in pre- and postmenopausal women by tumor hormone receptor status may be evident given differences in hormone metabolism and divergent associations between adiposity and breast cancer risk by menopausal status [1, 7]. A meta-analysis including both prospective cohorts and retrospective case–control studies, evaluated 4 studies with estrogen (ER) and progesterone (PR) receptor status and premenopausal breast cancer, and observed inverse associations for both ER+/PR+  and ER−/PR− disease [6]. Analyses by ER/PR status were not included in the recent UK Biobank study [3], and in the WCRF meta-analysis [1] analyses by hormone receptor status were limited to the more common outcome of postmenopausal breast cancer (ER +/PR+ , 6 studies and 5117 cases; ER−/PR−, 5 studies 1236 cases; restricted to prospective studies).

Given the relatively sparse data on physical activity in premenopausal women by hormone receptor subtype, we provide a comprehensive evaluation of recreational physical activity, overall and of moderate-vigorous intensity, and breast cancer risk by menopausal status and hormone receptor status in 187,278 women, including 12,785 breast cancer diagnoses, from two well-characterized cohorts with up to three decades of follow-up. The results of this study provide an update of prior findings from the Nurses’ Health Study (NHS) and NHSII, with more than double the number of cases and more than a decade of further follow-up [8, 9].

## Methods

### Study population: the Nurses’ Health Study (NHS) and Nurses’ Health Study II (NHSII)

The NHS was initiated in 1976 when 121,700 registered nurses in the United States, ages 30–55, completed and returned a mailed questionnaire [10, 11]. The NHSII was initiated in 1989 with 116,429 female registered nurses ages 25–42 and uses the same protocols. Participants in both cohorts complete mailed biennial questionnaires and provided updated information on lifestyle factors and disease diagnoses, including cancers. The studies were approved by the institutional review boards at Brigham and Women’s Hospital and Harvard T.H. Chan School of Public Health, and participating registries as required.

### Breast cancer case ascertainment

Participants reported disease status on the biennial NHS and NHSII questionnaires. Eligible cases reported no prior cancer diagnosis before baseline and were diagnosed with invasive breast cancer through June 2016 (NHS) or June 2017 (NHSII). A total of 12,785 eligible breast cancer cases were identified. A study physician confirmed cases through medical record review. Invasiveness*,* hormone receptor status, and tumor characteristics were abstracted from medical records. Vital status was ascertained through June 1, 2016 (NHS) or June 1, 2017 (NHSII) using next of kin reports, death certificates, and the National Death Index. Given the high confirmation rate by medical record for breast cancer in this cohort (99%) [12], we included both medical record and participant-confirmed cases in this study.

### Physical activity assessment

Recreational physical activity data was reported approximately every 4 years via self-administered questionnaire beginning in 1986 (NHS) and 1989 (NHSII). In the NHS, updated physical activity was reported in 1988, 1990 (walking only), 1992, 1994, 1996, 1998, 2000, 2004, 2006, 2008, 2010, 2012 and 2014 (2006, 2010: walking, jogging and running only). In the NHSII, physical activity was updated in 1991, 1997, 2001, 2005, 2009, and 2013.

Participants reported time spent (ten categories ranging from: 0 min/week to 11 + h/week), engaging in the following activities: walking or hiking outdoors; jogging (≥ 10 min/mile); running (< 10 min/mile); bicycling; calisthenics/aerobics; aerobic dance/rowing machine; tennis/squash/racquetball; lap swimming; and other aerobic recreation. Participants estimated walking pace (easy, casual (< 2 miles per hour (mph)); normal, average (2–2.9 mph); brisk (3–3.9 mph); very brisk/ striding (≥ 4 mph)). Energy expenditure was estimated by multiplying metabolic equivalent task (MET) values by reported duration for each activity [13] giving MET-h/week (e.g., 3–9 MET-h/week corresponds to approximately 1–3 h/week of walking at a pace of 2.5 mph).

Summary exposures included: total reported recreational physical activity (“total” activity in the current study refers to all recreational activities reported on the participant questionnaires) and moderate-vigorous activity (activities > 3 METs per hour: walking at pace ≥ 3 miles/hour, jogging, or running). Physical activity data was carried forward from the previous questionnaire year only in years when it was not queried (i.e., for 2002, activity reported in 2000 was used).

### Covariate assessment

Data on covariates was available from the baseline and/or biennial questionnaires. Age at each questionnaire was calculated using date of birth and questionnaire return date. Age at menarche and height were collected on the baseline questionnaires. Further covariates (date of collection) included: weight at age 18 (NHS: 1980; NHSII: 1989), weight (biennially), oral contraceptive use (OC; NHS: biennially until 1982; NHSII: biennially until 2009), menopausal hormone therapy use (HT; biennially), smoking status (biennially), alcohol consumption (every four years, NHS: from 1980; NHSII: from 1991), parity (NHS: biennially until 1984; NHSII: biennially), age at first birth (NHS: 1976 and biennially until 1982; NHS: 1989 and biennially), age at menopause (biennially), diagnosis of benign breast disease (biennially), and family history of breast cancer (NHS: every 4 years beginning in 1988; NHS2: 1989, and every 4 years beginning in 1997). Body mass index (BMI) at age 18 and current BMI were calculated using self-reported weight and height (BMI = weight in kilograms (kg)/height in meters (m)^2^).

### Statistical analysis

We calculated person-years beginning at date of baseline physical activity questionnaire return and ending at the earliest of date of diagnosis of any cancer (except non-melanoma skin cancer), death, or end of follow-up. We used multivariable Cox proportional hazards models to calculate hazard ratios (HR) using age in months as the time scale and stratified by follow-up year and cohort. Covariates were included in the models as time-varying covariates for variables with updated data throughout follow-up. Physical activity was evaluated using updated cumulative average over follow-up (i.e., average value across follow-up periods, with the average updated with each subsequent physical activity assessment). We used activity categories previously used in the cohorts (MET-h/week: < 3, 3 to < 9, 9 to < 18, 18 to < 27, ≥ 27). The reference category was < 3 MET-h/week; this level of activity corresponds to less than one hour of walking reported at “normal” or “average” pace per week. Tests for trend were evaluated by including category medians as continuous variables in the models. Covariates included in final models were: age at first birth and parity combined, birth index [14], age at menarche, BMI at age 18, menopausal status and age at menopause, oral contraceptive use, hormone therapy use, smoking, alcohol use, family history of breast cancer, and history of benign breast disease. Adjustment for current BMI was assessed in an additional model.

Associations were evaluated in strata of menopausal status and hormone receptor status. Heterogeneity (p_het_) in associations by hormone receptor status was assessed using a likelihood ratio test (LRT) comparing models assuming the same association between physical activity and breast cancer overall to one allowing different associations by receptor status using a competing risks model [15]. We evaluated heterogeneity in associations with overall, ER+/PR+ , and ER−/PR− disease by menopausal status (premenopausal, postmenopausal) by comparing models with and without an interaction term using the LRT. Within each menopausal group, we also evaluated heterogeneity by BMI category (< 25, ≥ 25 kg/m^2^). As a sensitivity analysis, we restricted analyses to women reporting a screening mammogram in the preceding two years to evaluate whether our results were impacted by differences in screening between physical activity subgroups (i.e., healthy behavior effect).

P values were considered statistically significant at < 0.05; all statistical tests are two-sided. Analyses were conducted in SAS 9.4 (Cary, NC).

## Results

Baseline characteristics were generally similar across physical activity categories in both cohorts (Table 1), though women reporting higher levels of physical activity at baseline were leaner (e.g., mean BMI in NHS, ≥ 27 vs. < 3 MET-h/week: 24.4 vs. 26.2 kg/m^2^). Average age at baseline was 52 years for NHS participants and 34 years for NHSII participants. A total of 39% of participants in the NHS were premenopausal at first physical activity assessment and 95% were parous, whereas 97% of participants in the NHSII were premenopausal and a lower proportion parous (e.g., parous, < 3 MET-h/week: 77%; ≥ 27: 65%). Median (interquartile range) reported activity at baseline was 7.7 MET-h/week (2.7–19.0) in the NHS and 13.7 MET-h/week (5.2–30.2) in the NHSII. Average activity at the first assessment across categories and ranged from e.g., 1.4 MET-h/week for individuals reporting < 3 MET-h/week of activity to 33.7 MET-h/week for women reporting ≥ 27 MET-h/week of activity in the NHS.

**Table 1:** Baseline characteristics at first recreational physical activity assessment: Nurses' Health Study (1986) and Nurses' Health Study II (1989)

Nurses' Health Study					
	Physical Activity in 1986 (MET-h/week)				
	< 3 (n = 21,830)	3- < 9 (n = 20,869)	9- < 18 (n = 14,919)	18- < 27 (n = 8263)	≥ 27 (n = 6453)
Age, 1986	52.2 (7.0)	52.4 (7.1)	52.4 (7.1)	52.4 (7.1)	52.6 (7.1)
Total Activity, MET-h/wk	1.4 (0.9)	5.4 (1.8)	12.9 (2.7)	22.1 (2.5)	34.0 (4.3)
BMI (current), kg/m^2^	26.2 (5.4)	25.6 (4.8)	25.0 (4.4)	24.6 (4.0)	24.4 (4.1)
BMI (age 18), kg/m^2^	21.5 (3.1)	21.3 (2.8)	21.3 (2.7)	21.3 (2.6)	21.3 (2.8)
Age at Menarche, Years	12.5 (1.4)	12.5 (1.4)	12.5 (1.4)	12.5 (1.4)	12.5 (1.4)
Parous, %	95	95	95	95	94
Parity (among parous women)	3.2 (1.5)	3.2 (1.5)	3.1 (1.5)	3.1 (1.4)	3.1 (1.5)
Age at First Birth, Years	25.2 (3.4)	25.1 (3.3)	25.0 (3.3)	25.0 (3.2)	25.0 (3.1)
Smoking, %					
Never	44	47	46	46	44
Past	31	33	36	38	39
Current	25	20	17	16	17
Family history of breast cancer, %	7	8	8	8	7
History of benign breast disease, %	33	34	35	36	35
Menopausal status, %					
Premenopausal	39	39	39	40	39
Postmenopausal	54	54	54	53	54
Perimenopausal/Unknown	7	7	7	7	7
Current HRT use, % (if postmenopausal)	23	25	27	29	29

Nurses' Health Study II					
	Physical Activity in 1989 (MET-h/week)				
	< 3 (n = 17,408)	3- < 9 (n = 26,020)	9- < 18 (n = 23,994)	18- < 27 (n = 15,079)	≥27 (n = 32,443)
Age, 1989	34.9 (4.6)	34.5 (4.6)	34.4 (4.6)	34.3 (4.6)	33.7 (4.8)
Total Activity, MET-h/wk	1.4 (0.90)	5.7 (1.7)	13.0 (2.6)	22.1 (2.5)	62.5 (51.0)
BMI, kg/m^2^	25.2 (6.1)	24.6 (5.3)	24.0 (4.8)	23.7 (4.4)	23.2 (4.3)
BMI (age 18), kg/m^2^	21.4 (3.6)	21.3 (3.3)	21.3 (3.3)	21.3 (3.2)	21.2 (3.3)
Age at Menarche, Years	12.9 (1.0)	12.9 (1.0)	12.9 (1.0)	12.9 (1.0)	13.0 (1.0)
Parous, %	77	75	73	69	65
Parity (among parous women)	2.1 (0.9)	2.1 (0.9)	2.1 (0.9)	2.1 (0.9)	2.1 (0.9)
Age at First Birth, Years	25.5 (4.1)	25.6 (4.0)	25.5 (3.9)	25.5 (4.0)	25.3 (4.1)
Smoking, %					
Never	65	66	66	66	65
Past	20	20	21	22	23
Current	16	14	12	12	12
Family History of Breast Cancer, %	6	6	5	6	6
History of Benign Breast Disease, %	8	8	8	8	8
Menopausal status, %					
Premenopausal	97	97	97	97	97
Postmenopausal	2	2	2	2	2
Perimenopausal/Unknown	1	1	1	1	1
Current HT use, % (postmenopausal)	86	91	90	87	87

Values are means (SD) or percentages and are standardized to the age distribution of the study population

Values of polytomous variables may not sum to 100% due to rounding

3–9 MET-h/week corresponds to approximately 1–3 h/week of walking at a pace of 2.5 mph

*Value is not age adjusted

Total reported recreational physical activity was inversely associated with overall breast cancer risk in pre- and postmenopausal women (premenopausal: HR = 0.91, 95%CI = (0.80–1.04); postmenopausal, 0.87 (0.80–0.94)). Recreational physical activity was inversely associated with ER+/PR+  breast cancer risk, with similar associations observed in both pre- and postmenopausal women (≥ 27 vs < 3 MET-h/week, premenopausal HR = 0.83, 95%CI = (0.70–0.99), p_trend_ = 0.06; postmenopausal HR = 0.86 (0.78–0.95), p_trend_ < 0.01; p_heterogeneity_ = 0.97) (Table 2). The association in premenopausal women was unchanged with adjustment for current BMI (≥ 27 vs < 3 MET-h/week, 0.83 (0.69–0.98), p_trend_ = 0.05), while the association in postmenopausal women was attenuated after adjustment (0.95 (0.85–1.05), p_trend_ = 0.09). We observed limited evidence of heterogeneity in the association between moderate-vigorous activity and ER+/PR+  breast cancer risk by menopausal status (p_het_ ≥ 0.58; e.g., ≥ 27 vs < 3 MET-h/week, premenopausal HR = 0.88 (0.69–1.11); postmenopausal HR = 0.71 (0.58–0.88)). No associations were observed for ER−/PR− breast cancer. Among women reporting a mammogram in the prior 2 years associations with total and moderate-vigorous activity were similar to the overall analyses (Table S1).

**Table 2:** Association between overall and moderate-vigorous recreational physical activity and risk of breast cancer by tumor hormone receptor status and menopausal status: NHS (1986–2016) and NHSII (1989–2017)

Premenopausal	MET-h/week									p_trend_	p_het ERPR_	P_int meno_
	< 3	3 to < 9		9 to < 18		19 to < 27		≥ 27				
	HR (95% CI)	HR (95% CI)		HR (95% CI)		HR (95% CI)		HR (95% CI)				
Overall recreational physical activity												
All cases (n = 2,935)												
n cases/person-years	352/210158	754/437474		712/460348		449/282961		668/472417				
Multivariable	Ref	1.02	(0.90–1.17)	0.91	(0.80–1.00)	0.95	(0.82–1.10)	0.91	(0.80–1.04)	0.06		0.90
Multivariable + current BMI	Ref	1.02	(0.89–1.16)	0.91	(0.80–1.04)	0.95	(0.82–1.10)	0.91	(0.79–1.04)	0.05		0.93
ER+/PR+ (n = 1,729)												
n cases/person-years	212/210158	435/437474		420/460348		274/282961		388/472417				
Multivariable	Ref	0.94	(0.79–1.11)	0.85	(0.72–1.01)	0.91	(0.76–1.09)	0.83	(0.70–0.99)	0.06	0.56	0.97
Multivariable + current BMI	Ref	0.94	(0.79–1.11)	0.85	(0.71–1.00)	0.90	(0.75–1.09)	0.83	(0.69–0.98)	0.05	0.52	0.99
ER−/PR− (n = 372)												
n cases/person-years	39/210158	98/437474		98/460348		60/282961		77/472417				
Multivariable	Ref	1.21	(0.83–1.77)	1.18	(0.80–1.72)	1.21	(0.80–1.82)	1.00	(0.67–1.48)	0.48		0.47
Multivariable + current BMI	Ref	1.22	(0.84–1.78)	1.19	(0.81–1.74)	1.23	(0.81–1.86)	1.03	(0.69–1.53)	0.59		0.46
Moderate-vigorous activity												
All cases (n = 2935)												
n cases/person-years	1772/1131584	584/346409		327/203911		128/85241		124/96215				
Multivariable	Ref	0.98	(0.89–1.08)	0.95	(0.84–1.06)	0.94	(0.78–1.12)	0.85	(0.71–1.03)	0.06		0.82
Multivariable + current BMI	Ref	0.98	(0.89–1.08)	0.94	(0.84–1.06)	0.93	(0.78–1.12)	0.85	(0.71–1.02)	0.06		0.90
ER+/PR+ (n = 1,729)												
n cases/person-years	1028/1131584	362/346409		184/203911		81/85241		74/96215				
Multivariable	Ref	1.01	(0.90–1.14)	0.89	(0.76–1.04)	1.02	(0.81–1.28)	0.88	(0.69–1.11)	0.23	0.76	0.58
Multivariable + current BMI	Ref	1.01	(0.89–1.14)	0.88	(0.75–1.04)	1.01	(0.81–1.27)	0.87	(0.68–1.11)	0.19	0.75	0.68
ER−/PR− (n = 372)												
n cases/person-years	226/1131584	73/346409		42/203911		20/85241		11/96215				
Multivariable	Ref	1.02	(0.78–1.34)	1.02	(0.73–1.42)	1.18	(0.74–1.87)	0.65	(0.35–1.19)	0.52		0.55
Multivariable + current BMI	Ref	1.04	(0.79–1.36)	1.04	(0.75–1.46)	1.21	(0.76–1.92)	0.67	(0.36–1.23)	0.63		0.53

Postmenopausal												
Overall recreational physical activity												
All cases (n = 9850)												
n cases/person-years	1153/345134	2551/779156		2773/864669		1557/504052		1816/657671				
Multivariable	Ref	0.98	(0.91–1.05)	0.96	(0.89–1.03)	0.93	(0.86–1.01)	0.87	(0.80–0.94)	< 0.01		
Multivariable + current BMI	Ref	1.00	(0.93–1.08)	1.00	(0.93–1.07)	0.99	(0.91–1.07)	0.94	(0.87–1.01)	0.04		
ER+/PR+ (n = 5598)												
n cases/person-years	621/345144	1432/779163		1648/864649		886/504052		1011/657671				
Multivariable	Ref	0.99	(0.90–1.09)	1.01	(0.92–1.11)	0.93	(0.84–1.03)	0.86	(0.78–0.95)	< 0.01	0.02	
Multivariable + current BMI	Ref	1.02	(0.93–1.12)	1.06	(0.97–1.17)	1.00	(0.90–1.11)	0.95	(0.85–1.05)	0.09	0.05	
ER−/PR− (n = 1106)												
n cases/person-years	113/345144	291/779163		283/864649		206/504052		213/657671				
Multivariable	Ref	1.13	(0.90–1.41)	1.01	(0.80–1.26)	1.27	(1.00–1.61)	1.05	(0.83–1.33)	0.64		
Multivariable + current BMI	Ref	1.14	(0.91–1.42)	1.02	(0.81–1.28)	1.29	(1.02–1.64)	1.08	(0.85–1.37)	0.49		
Moderate-Vigorous Activity												
All cases (n = 9850)												
n cases/person-years	6356/1956010	1918/641259		1055/349125		333/118321		188/85966				
Multivariable adjusted	Ref	0.95	(0.90–1.00)	0.96	(0.89–1.02)	0.89	(0.79–0.99)	0.76	(0.66–0.88)	< 0.01		
Multivariable adjusted + current BMI	Ref	0.99	(0.94–1.04)	1.01	(0.95–1.08)	0.95	(0.85–1.07)	0.83	(0.72–0.96)	0.06		
ER+/PR+ (n = 5598)												
n cases/person-years	3575/1956010	1136/641259		615/349125		172/118321		100/85966				
Multivariable	Ref	0.98	(0.91–1.05)	0.97	(0.89–1.06)	0.81	(0.70–0.95)	0.71	(0.58–0.88)	< 0.01	0.22	
Multivariable + current BMI	Ref	1.03	(0.96–1.10)	1.05	(0.96–1.14)	0.89	(0.76–1.04)	0.80	(0.65–0.98)	0.10	0.40	
ER−/PR− (n = 1106)												
n cases/person-years	691/1956010	212/641259		131/349125		44/118321		28/85966				
Multivariable	Ref	0.93	(0.79–1.09)	1.04	(0.86–1.26)	1.07	(0.79–1.46)	1.05	(0.70–1.50)	0.65		
Multivariable + current BMI	Ref	0.94	(0.80–1.11)	1.06	(0.87–1.28)	1.09	(0.80–1.50)	1.05	(0.72–1.54)	0.50		

Multivariable models adjusted for age at first birth and parity (nulliparous, 1–2 birth ≤ 25y, 1–2 births > 25 y, 3 + births ≤ 25, 3 + births > 25y), birth index, age at menarche (categorical), family history of breast cancer, history of benign breast disease, oral contraceptive use (categorical, premenopausal only), current smoking status, alcohol (categories), hormone therapy use (categorical, postmenopausal only), age at menopause (postmenopausal only), BMI at age 18 (continuous)

Note: 3–9 MET-h/week corresponds to approximately 1–3 h/week of walking at a pace of 2.5 mph

We evaluated heterogeneity in associations by BMI separately in pre- (Table 3) and postmenopausal women (Table 4). In premenopausal women, associations comparing highest to lowest activity subgroup were similar in both BMI subgroups (e.g., total activity, ER+/PR+, ≥ 27 vs. < 3 MET-h/week, BMI < 25: 0.84 (0.66–1.08) p_trend_ = 0.01; ≥ 25: 0.86 (0.67–1.11) p_trend_ = 0.99) (Table 3). Among postmenopausal women, inverse associations between total activity and ER+/PR+  breast cancer were only observed in women with BMI ≥ 25 kg/m^2^ (e.g., ≥ 27 vs. < 3 MET-h/week, BMI ≥ 25: 0.80 (0.69–0.92) p_trend_ < 0.001; < 25: 0.96 (0.80–1.15) p_trend_ = 0.74) (Table 4). Moderate-vigorous activity results were similar in both strata of BMI among postmenopausal women.

**Table 3:** Association between overall and moderate-vigorous recreational physical activity and risk of breast cancer by tumor hormone receptor status and BMI (< 25, ≥ 25 kg/m^2^): NHS (1986–2016) and NHSII (1989–2017) among premenopausal women

	MET-h/week											
	< 3	3 to < 9		9 to < 18		19 to < 27		≥ 27		p_trend_	p_het ERPR_	p_int BMI_^†^
	HR (95% CI)	HR (95% CI)		HR (95% CI)		HR (95% CI)		HR (95% CI)				
BMI < 25 kg/m^2^												
Overall recreational physical activity												
All cases (n = 1599)												
n cases/person-years	159/100296	385/217761		398/247183		248/161708		409/302046				
Multivariable	Ref	1.12	(0.93–1.35)	1.02	(0.85–1.23)	0.97	(0.79–1.19)	0.92	(0.76–1.11)	0.02		0.52
Multivariable + current BMI	Ref	1.12	(0.93–1.35)	1.02	(0.85–1.23)	0.97	(0.97–1.19)	0.92	(0.76–1.11)	0.02		0.54
ER+/PR+ (n = 932)												
n cases/person-years	93/100296	232/217761		227/247183		149/161708		231/302046				
Multivariable	Ref	1.11	(0.87–1.42)	0.94	(0.74–1.21)	0.94	(0.72–1.22)	0.84	(0.66–1.08)	0.01	0.25	0.33
Multivariable + current BMI	Ref	1.11	(0.87- 1.42)	0.94	(0.74–1.21)	0.94	(0.72–1.22)	0.84	(0.65–1.08)	0.01	0.26	0.36
ER−/PR− (n = 194)												
n cases/person-years	14/100296	48/217761		59/247183		31/ 161708		42/302046				
Multivariable	Ref	1.60	(0.88–2.93)	1.79	(0.99–3.24)	1.46	(0.76–2.77)	1.10	(0.59–2.05)	0.20		0.60
Multivariable + current BMI	Ref	1.60	(0.88–2.93)	1.79	(0.99–3.25)	1.46	(0.77–2.79)	1.12	(0.60–2.08)	0.23		0.56
Moderate-Vigorous Activity												
All cases (n = 1599)												
n cases/person-years	874/576024	359/198532		196/125932		77/56437		93/72070				
Multivariable	Ref	1.06	(0.93–1.20)	0.93	(0.80–1.09)	0.85	(0.67–1.08)	0.84	(0.68–1.05)	0.04		0.73
Multivariable + current BMI	Ref	1.06	(0.93–1.20)	0.93	(0.80–1.09)	0.85	(0.67–1.08)	0.85	(0.57–1.05)	0.04		0.74
ER+/PR+ (n = 932)												
n cases/person-years	509/576024	213/198532		108/125932		48/56437		54/72070				
Multivariable	Ref	1.04	(0.89–1.23)	0.85	(0.69–1.05)	0.90	(0.67–1.22)	0.83	(0.62–1.11)	0.08	0.48	0.54
Multivariable + current BMI	Ref	1.04	(0.89–1.23)	0.85	(0.69–1.05)	0.90	(0.67–1.22)	0.83	(0.62–1.11)	0.08	0.50	0.58
ER−/PR− (n = 194)												
n cases/person-years	105/576024	49/198532		25/125932		7/56437		8/72070				
Multivariable	Ref	1.28	(0.91–1.81)	1.08	(0.69–1.68)	0.66	(0.30–1.42)	0.65	(0.31–1.33)	0.16		0.16
Multivariable + current BMI	Ref	1.29	(0.91–1.82)	1.08	(0.70–1.69)	0.66	(0.31–1.43)	0.66	(0.32–1.36)	0.18		0.18
BMI ≥ 25 kg/m2												
Overall recreational physical activity												
All cases (n = 1316)												
n cases/person-years	190/109403	367/217305		310/209221		197/118445		252/165356				
Multivariable	Ref	0.94	(0.78–1.12)	0.82	(0.68–0.99)	0.94	(0.77–1.16)	0.94	(0.78–1.15)	0.91		
Multivariable + current BMI	Ref	0.94	(0.78–1.12)	0.82	(0.68–0.99)	0.94	(0.76–1.15)	0.94	(0.77–1.14)	0.88		
ER+/PR+ (n = 781)												
n cases/person-years	117/109403	202/217305		190/209221		121/118445		151/165356				
Multivariable	Ref	0.80	(0.64–1.01)	0.78	(0.62–0.99)	0.88	(0.68–1.15)	0.86	(0.67–1.11)	0.99	0.90	
Multivariable + current BMI	Ref	0.80	(0.63–1.01)	0.78	(0.61–0.99)	0.88	(0.67–1.14)	0.86	(0.66–1.10)	0.94	0.88	
ER−/PR− (n = 178)												
n/person-years	25/109403	50/217305		39/209221		29/118445		35/165356				
Multivariable	Ref	0.96	(0.59–1.56)	0.78	(0.46–1.30)	1.08	(0.62–1.88)	1.03	(0.61–1.74)	0.65		
Multivariable + current BMI	Ref	0.97	(0.60–1.58)	0.79	(0.47–1.33)	1.11	(0.64–1.93)	1.06	(0.62–1.80)	0.57		
Moderate-Vigorous Activity												
All cases (n = 1316)												
n cases/person-years	886/549231	223/143772		128/75403		50/27961		29/23364				
Multivariable	Ref	0.88	(0.76–1.03)	0.99	(0.82–1.19)	1.12	(0.84–1.49)	0.84	(0.58–1.22)	0.72		
Multivariable + current BMI	Ref	0.88	(0.76–1.02)	0.98	(0.81–1.19)	1.12	(0.84–1.49)	0.83	(0.57–1.21)	0.69		
ER+/PR+ (n = 781)												
n cases/person-years	510/549231	147/143772		74/75403		32/27961		18/23364				
Multivariable	Ref	0.97	(0.80–1.17)	0.95	(0.74–1.22)	1.25	(0.87–1.79)	0.89	(0.55–1.43)	0.92	0.32	
Multivariable + current BMI	Ref	0.97	(0.80–1.17)	0.94	(0.74–1.21)	1.24	(0.86–1.77)	0.88	(0.55–1.42)	0.97	0.32	
ER−/PR− (n = 178)												
n cases/person-years	121/549231	24/ 143,772		17/75403		13/27961		3/23364				
Multivariable	Ref	0.75	(0.48–1.16)	1.02	(0.61–1.71)	2.21	(1.24–3.96)	0.66	(0.21–2.10)	0.35		
Multivariable + current BMI	Ref	0.76	(0.48–1.19)	1.05	(0.63–1.76)	2.27	(1.26–4.08)	0.69	(0.22–2.17)	0.29		

Multivariable models adjusted for age at first birth and parity (nulliparous, 1–2 birth ≤ 25y, 1–2 births > 25 y, 3 + births ≤ 25, 3 + births > 25y), birth index, age at menarche (categorical), family history of breast cancer, history of benign breast disease, oral contraceptive use (categorical), current smoking status, alcohol (categories), BMI at age 18 (continuous)

^†^ p value for heterogeneity tests model with BMI (high v. low) in place of continuous BMI vs. interaction model

Note: 3–9 MET-h/week corresponds to approximately 1–3 h/week of walking at a pace of 2.5 mph

**Table 4:** Association between overall and moderate-vigorous recreational physical activity and risk of breast cancer by tumor hormone receptor status and BMI (< 25, ≥ 25 kg/m^2^): NHS (1986–2016) and NHSII (1989–2017) among postmenopausal women

	MET-h/week											
	< 3	3 to < 9		9 to < 18		19 to < 27		≥ 27		p_trend_	p_het ERPR_	p_int BMI_^†^
	HR (95% CI)	HR (95% CI)		HR (95% CI)		HR (95% CI)		HR (95% CI)				
BMI < 25 kg/m^2^												
Overall recreational physical activity												
All cases (n = 3573)												
n cases/person-years	347/105553	800/242395		967/294278		598/188820		861/281187				
Multivariable	Ref	0.97	(0.85–1.11)	0.95	(0.84–1.08)	0.94	(0.82–1.08)	0.96	(0.84–1.09)	0.52		0.10
Multivariable + current BMI	Ref	0.97	(0.85–1.10)	0.95	(0.84–1.08)	0.94	(0.82–1.08)	0.96	(0.85–1.10)	0.69		0.27
ER+/PR+ (n = 1938)												
n cases/person-years	179/105553	419/242395		549/294278		311/188820		480/281187				
Multivariable	Ref	0.93	(0.78–1.11)	0.96	(0.81–1.14)	0.87	(0.72–1.05)	0.96	(0.80–1.15)	0.74	0.15	0.09
Multivariable + current BMI	Ref	0.92	(0.77–1.10)	0.96	(0.80–1.14)	0.87	(0.72–1.05)	0.97	(0.81–1.16)	0.93	0.15	0.27
ER−/PR− (n = 455)												
n cases/person-years	42/105553	98/242395		115/294278		95/188820		105/281187				
Multivariable	Ref	0.98	(0.68–1.43)	1.00	(0.69–1.44)	1.29	(0.88–1.90)	1.02	(0.70–1.48)	0.46		0.88
Multivariable + current BMI	Ref	0.98	(0.68–1.43)	1.00	(0.69–1.44)	1.30	(0.88–1.90)	1.02	(0.70–1.49)	0.45		0.91
Moderate-Vigorous Activity												
All cases (n = 3573)												
n cases/person-years	2019/611222	774/243069		490/152195		176/58598		114/ 47148				
Multivariable	Ref	0.97	(0.89–1.06)	0.99	(0.90–1.10)	0.94	(0.81–1.11)	0.83	(0.69–1.01)	0.10		0.73
Multivariable + current BMI	Ref	0.98	(0.89–1.06)	1.00	(0.90–1.11)	0.96	(0.82–1.12)	0.85	(0.70–1.04)	0.18		0.98
ER+/PR+ (n = 1938)												
n cases/person-years	1062/611222	450/243069		281/152195		84/58598		61/ 47148				
Multivariable	Ref	1.03	(0.92–1.15)	1.06	(0.93–1.22)	0.85	(0.68–1.07)	0.84	(0.64–1.09)	0.21	0.16	0.64
Multivariable + current BMI	Ref	1.03	(0.92–1.16)	1.07	(0.94–1.23)	0.87	(0.69–1.09)	0.87	(0.67–1.14)	0.35	0.19	0.95
ER−/PR− (n = 455)												
n cases/person-years	255/611222	93/243069		58/152195		28/58598		21/47148				
Multivariable	Ref	0.93	(0.72–1.19)	0.93	(0.69–1.26)	1.27	(0.85–1.88)	1.25	(0.80–1.97)	0.26		0.83
Multivariable + current BMI	Ref	0.93	(0.73–1.19)	0.93	(0.69–1.26)	1.27	(0.85–1.89)	1.26	(0.80–1.97)	0.25		0.88
BMI ≥ 25 kg/m2												
Overall recreational physical activity												
All cases (n = 5317)												
n cases/person-years	747/205142	1550/425769		1502/426489		772/225847		746/253964				
Multivariable	Ref	0.99	(0.90–1.08)	0.95	(0.87–1.04)	0.94	(0.85–1.04)	0.83	(0.75–0.93)	< 0.001		
Multivariable + current BMI	Ref	1.00	(0.92–1.10)	0.97	(0.89–1.07)	0.97	(0.87–1.08)	0.87	(0.78–0.96)	0.003		
ER+/PR+ (n = 3061)												
n cases/person-years	409/205142	897/425769		891/426489		460/225847		404/253964				
Multivariable	Ref	1.02	(0.90–1.15)	0.99	(0.87–1.11)	0.97	(0.84–1.11)	0.80	(0.69–0.92)	< 0.001	0.07	
Multivariable + current BMI	Ref	1.04	(0.92–1.17)	1.02	(0.90–1.15)	1.01	(0.88–1.16)	0.84	(0.73–0.97)	0.005	0.09	
ER−/PR− (n = 569)												
n cases/person-years	67/205142	178/425769		138/426489		98/225847		88/253964				
Multivariable	Ref	1.24	(0.93–1.65)	0.95	(0.70–1.29)	1.30	(0.94–1.80)	1.07	(0.77–1.49)	0.94		
Multivariable + current BMI	Ref	1.25	(0.93–1.67)	0.97	(0.71–1.31)	1.33	(0.96–1.84)	1.10	(0.78–1.53)	0.82		
Moderate-Vigorous Activity												
All cases (n = 5317)												
n cases/person-years	3782/1056710	901/278030		446/135321		122/41641		66/25509				
Multivariable	Ref	0.94	(0.88–1.02)	0.96	(0.87–1.06)	0.86	(0.72–1.04)	0.88	(0.69–1.12)	0.04		
Multivariable + current BMI	Ref	0.97	(0.90–1.04)	0.99	(0.89–1.09)	0.89	(0.74–1.07)	0.91	(0.71–1.17)	0.21		
ER+/PR+ (n = 3061)												
n cases/person-years	2169/1056710	528/278030		265/135321		64/41641		35/25509				
Multivariable	Ref	0.94	(0.85–1.04)	0.98	(0.86–1.11)	0.78	(0.60–1.00)	0.81	(0.57–1.14)	0.04	0.65	
Multivariable + current BMI	Ref	0.97	(0.88–1.07)	1.02	(0.89–1.16)	0.81	(0.63–1.05)	0.85	(0.61–1.20)	0.18	0.74	
ER−/PR− (n = 569)												
n cases/person-years	385/1056710	105/278030		58/135321		15/41641		6/25509				
Multivariable	Ref	1.01	(0.81–1.27)	1.12	(0.84–1.49)	0.97	(0.56–1.67)	0.77	(0.34–1.72)	0.97		
Multivariable + current BMI	Ref	1.03	(0.82–1.29)	1.14	(0.85–1.52)	0.99	(0.57–1.71)	0.79	(0.35–1.77)	0.92		

Multivariable models adjusted for age at first birth and parity (nulliparous, 1–2 birth ≤ 25y, 1–2 births > 25 y, 3 + births ≤ 25, 3 + births > 25y), birth index, age at menarche (categorical), family history of breast cancer, history of benign breast disease, current smoking status, alcohol (categories), hormone therapy use (categorical), age at menopause, BMI at age 18 (continuous)

^†^ p value for heterogeneity tests model with BMI (high v. low) in place of continuous BMI vs. interaction model

Note: 3–9 MET-h/week corresponds to approximately 1–3 h/week of walking at a pace of 2.5 mph

## Discussion

Higher levels of recreational physical activity were associated with lower risk of hormone receptor-positive breast cancer in this large prospective study. These findings provide support for a role for physical activity in primary prevention of breast cancer. Inverse associations were observed regardless of menopausal status, though while associations for premenopausal disease were robust to adjustment for BMI, associations for postmenopausal disease were attenuated by BMI adjustment. This study adds to the relatively sparse literature on recreational physical activity and breast cancer in premenopausal women by hormone receptor status, observing similar inverse associations in pre- and postmenopausal women.

Prior studies have reported lower risk of breast cancer with higher levels of total recreational physical activity, with a recent meta-analysis of prospective studies noting an inverse association of similar magnitude in pre- and postmenopausal women (e.g., “high” vs. “low” recreational activity, premenopausal, relative risk (RR) = 0.89 (0.74–1.04); postmenopausal, 0.88 (0.82–0.94)) [1]. A limited number of studies with results by hormone receptor status precluded meta-analyses by hormone receptor status among premenopausal women. A lower risk of ER+/PR+  postmenopausal breast cancer was reported with higher recreational activity (n = 6 studies, 5117 cases, RR = 0.89 (0.82–0.96)), and the association ER−/PR− breast cancer was of the same magnitude (n = 5 studies, 1236 cases, 0.89 (0.76–1.04)) [1]. We observed heterogeneity when examining overall physical activity and breast cancer risk by hormone receptor status only among postmenopausal women; however, in both pre- and postmenopausal women, associations were only observed for ER+/PR+  disease. Heterogeneity by hormone receptor status has largely not been observed in prior studies of physical activity and breast cancer risk [8, 16–20], though associations are more consistently observed for hormone responsive disease. The current study includes more postmenopausal ER+/PR+  cases (n = 5598) than the prior published meta-analysis [1], and a substantial number of ER−/PR− cases (n = 1106), and adds needed data on physical activity and breast cancer in premenopausal women (n = 1729 ER+/PR+, n = 372 ER−/PR−).

Findings from prior studies have suggested vigorous activity may have differential effects on breast cancer risk by menopausal status, with a prior meta-analysis of prospective studies [1] reporting a suggestively stronger inverse association for vigorous activity among premenopausal women, (“high” vs. “low”, premenopausal, 0.79 (0.74–1.04); postmenopausal, 0.90 (0.85–0.95)). A separate meta-analysis [6] on moderate-vigorous activity including both retrospective and prospective studies observed lower risk of ER+/PR+  and ER−/PR− in premenopausal women, as compared to postmenopausal women (e.g., ER+/PR+: premenopausal, 4 studies, RR = 0.60 (0.45–0.81); postmenopausal, 13 studies, 0.79 (0.71–0.89)). We did not observe this pattern for moderate-vigorous activity in the current study, with a somewhat stronger association observed in postmenopausal women (ER+/PR+, ≥ 27 vs. < 3 MET-h/week, 0.71 (0.58–0.88)) and an inverse association observed in premenopausal women (0.88 (0.69–1.11)); associations in the current study were similar when considering all cases (i.e., regardless of ER/PR status).

We observed limited heterogeneity in associations by BMI in the current study, although the inverse association between total recreational activity and risk in the current study was predominantly observed in postmenopausal women with BMI ≥ 25 kg/m^2^. This is in contrast to a meta-analysis evaluating total activity, which reported a stronger association among postmenopausal normal-weight women [1]. A recent study in the UK Biobank [3] observed no heterogeneity in associations by BMI, with similar associations in strata of BMI and an inverse association among (pre- and postmenopausal) women with BMI ≥ 25 kg/m^2^. We observed that the associations between moderate-vigorous activity and breast cancer risk were similar in both strata of BMI. This is consistent with a meta-analysis on moderate-vigorous activity which reported similar associations in strata of BMI [6], though this was only observed for postmenopausal women.

Physical activity may influence breast cancer risk through its impact on adiposity, and adiposity-related mechanisms including sex steroid hormone metabolism, and inflammation and immune-related pathways. The associations between adiposity [21, 22] and physical activity [23, 24] and these mechanisms have been described previously with lower levels of adiposity and higher levels of physical activity, for example, associated with lower circulating estradiol, improvements in insulin resistance, and lower concentrations of inflammation marker C-reactive protein, and altered immune response.

We adjusted for current BMI in secondary models given that BMI may be on the causal pathway. BMI adjustment had essentially no impact on the association between physical activity and breast cancer in premenopausal women, but attenuated associations for ER+/PR+  postmenopausal disease (e.g., total activity unadjusted HR, 0.86 (0.78–0.95); adjusted, 0.95 (0.85–1.05)). The associations for postmenopausal breast cancer are in line with the well-established positive association between BMI and postmenopausal breast cancer [1]. In a recent study in the UK Biobank, accelerometer-based physical activity was evaluated, and analyses were adjusted for fat mass measured with bioimpedance [3]. Small changes in the associations between physical activity and breast cancer risk were noted after adjustment for body fatness, in the direction of strengthening associations in premenopausal women (RR per 5 milligravity units of activity, before adjustment, 0.82 (0.69–0.97); after adjustment, 0.79 (0.66–0.95)) and attenuating associations after adjustment in postmenopausal women (before adjustment, 0.79 (0.69–0.90); after adjustment, 0.84 (0.73–0.96)). Notably, BMI is associated with a lower risk of breast cancer in premenopausal women [7], likely explaining why this adjustment did not impact findings for physical activity and pre-menopausal breast cancer. It is plausible that the associations between physical activity and premenopausal breast cancer are underpinned by direct effects of physical activity on intermediate mechanistic pathways, and that associations are independent of adiposity.

Our study has strengths and limitations. The biennial follow-up enabled us to update physical activity and covariate data throughout the study period, and we used a cumulative average measure to represent habitual physical activity across follow-up. A limitation of using the cumulative average physical activity measure, together with time-varying covariates, is the potential conditioning on mediating variables across follow-up (i.e., those variables impacted by past activity as reflected in the cumulative average). Results from age-adjusted models were similar to those from the multivariable models, though with less precise confidence intervals, thus we do not expect this has had a substantial impact on our results. The BMI-adjusted models would be expected to be the most substantially impacted by this issue, and this may have led to an attenuation of the effect of adjustment for BMI. A limitation of the study is the use of self-reported activity, however, this approach has demonstrated reliability and validity [25] and has been associated with other outcomes [26–29]. The levels of recreational activity reported in the NHS and NHSII are similar to those reported in other prospective cohorts [30]. Further, our results describe associations with recreational, discretionary activity and not occupational or household and caregiving physical activity. Recreational activity may represent activity types that are most amenable to change, and thus with potential for intervention toward cancer prevention. However, lack of data on other activity types precludes an evaluation of total physical activity across domains. The results of this updated study within the NHS and NHSII are in line with earlier findings from these cohorts with shorter follow-up [8, 9]. Finally, while this study adds needed information on physical activity and breast cancer risk in premenopausal women by tumor hormone receptor status, sample size precluded an evaluation of more detailed tumor subtypes.

Higher levels of recreational physical activity were associated with lower risk of both pre- and postmenopausal hormone receptor-positive breast cancer, providing further support for the beneficial role of physical activity for cancer prevention. These results were robust to adjustment for current BMI in premenopausal women, whereas BMI adjustment attenuated the results in postmenopausal women, findings that are consistent with the established associations between adiposity-related mechanisms and postmenopausal breast cancer. These findings support recreational physical activity as a modifiable exposure associated with reduced breast cancer risk regardless of menopausal status.

### Supplementary Information

Below is the link to the electronic supplementary material.

Supplementary file1 (DOCX 37 kb)

## Data Availability

Data used in this study are available by application at nurseshealthstudy.org.
