# Supplementary material for: Recreational physical activity and breast cancer risk by menopausal status and tumor hormone receptor status: results from the Nurses’ Health Studies
Source: Breast Cancer Res Treat. 2024 Apr 9;206(1):77–90. doi: 10.1007/s10549-023-07238-x (PMC11182805; doi:10.1007/s10549-023-07238-x)
Supplement: Supplementary file 1 — Supplementary file1 (DOCX 37 kb) [file 10549_2023_7238_MOESM1_ESM.docx]

1

| **Table S1. Association between overall and moderate/vigorous physical activity and risk of breast cancer for women with mammogram in last 2 years: NHS (1986-2016) and NHSII (1989-2017)** | | | | | | | | | | | | | | |
| --- | --- | --- | --- | --- | --- | --- | --- | --- | --- | --- | --- | --- | --- | --- |
|  | **MET-hrs/week** | | | | | | | | | |  |  |  |  |
|  | **<3** | | **3 - <9** | | **9 - <18** | | **19 - <27** | | **≥27** | |  |  | |  |
|  | **HR (95% CI)** | | **HR (95% CI)** | | **HR (95% CI)** | | **HR (95% CI)** | | **HR (95% CI)** | | **p_trend_** | **p_int_** | |  |
| **Premenopausal** |  |  |  |  |  |  |  |  |  |  |  |  | |  |
| **Overall physical activity** |  |  |  |  |  |  |  |  |  |  |  |  | |  |
| **All cases (n=1,953)** |  |  |  |  |  |  |  |  |  |  |  |  | |  |
| **n/person-years** | 220/ 106624 | | 497/230846 | | 481/ 250144 | | 317/153364 | | 438/235255 | |  |  | |  |
| Multivariable | ref | | 1.03 | (0.88-1.22) | 0.92 | (0.78-1.09) | 1.00 | (0.83-1.19) | 0.91 | (0.77-1.08) | 0.14 | 0.87 | |  |
| Multivariable + current BMI | ref | | 1.03 | (0.88-1.22) | 0.92 | (0.78-1.09) | 0.99 | (0.83-1.19) | 0.91 | (0.77-1.08) | 0.14 | 0.90 | |  |
| **Moderate/Vigorous Activity (>3 METs/hour)** | | |  |  |  |  |  |  |  |  |  |  | |  |
| **All cases (n=1,953)** |  | |  | |  | |  | |  | |  |  | |  |
| **n/person-years** | 1151/571141 | | 420/199586 | | 227/114665 | | 83/45598 | | 72/45243 | |  |  | |  |
| Multivariable | ref | | 1.00 | (0.89-1.12) | 0.93 | (0.81-1.07) | 0.88 | (0.71-1.11) | 0.77 | (0.60-0.98) | 0.02 | 0.94 | |  |
| Multivariable + current BMI | ref | | 1.00 | (0.89-1.12) | 0.93 | (0.80-1.07) | 0.88 | (0.70-1.1) | 0.77 | (0.60-0.98) | 0.02 | 0.89 | |  |
| **Postmenopausal** |  | |  |  |  |  |  |  |  |  |  |  | |  |
| **Overall physical activity** |  | |  |  |  |  |  |  |  |  |  |  | |  |
| **All cases (n=7,315)** |  | |  |  |  |  |  |  |  |  |  |  | |  |
| **n/person-years** | 738/195998 | | 1874/498354 | | 2090/573891 | | 1217/336333 | | 1396/420432 | |  |  | |  |
| Multivariable | ref | | 0.96 | (0.88-1.05) | 0.91 | (0.84-1.00) | 0.91 | (0.83-1.00) | 0.85 | (0.77-0.93) | <0.001 |  | |  |
| Multivariable + current BMI | ref | | 0.98 | (0.90-1.07) | 0.95 | (0.87-1.04) | 0.96 | (0.88-1.06) | 0.92 | (0.83-1.00) | 0.04 |  | |  |
| **Moderate/Vigorous Activity (>3 METs/hour)** | | | | | | | | | | | |  | |  |
| **All cases (n=7,315)** |  | |  |  |  |  |  |  |  |  |  |  | |  |
| **n/person-years** | 4555/1226222 | | 1540/434869 | | 813/234330 | | 260/77812 | | 147/51775 | |  |  | |  |
| Multivariable | ref | | 0.95 | (0.90-1.01) | 0.92 | (0.86-1.00) | 0.89 | (0.79-1.01) | 0.81 | (0.69-0.96) | 0.001 |  | |  |
| Multivariable + current BMI | ref | | 0.99 | (0.93-1.05) | 0.98 | (0.90-1.05) | 0.96 | (0.84-1.09) | 0.89 | (0.75-1.05) | 0.13 |  | |  |
| Multivariable models adjusted for age at first birth and parity (nulliparous, 1-2 birth ≤25y, 1-2 births >25 y, 3+ births ≤25, 3+births >25y), birth index, age at menarche (categorical), family history of breast cancer, history of benign breast disease, oral contraceptive use (categorical, premenopausal only), current smoking status, alcohol (categories), hormone therapy use (categorical, postmenopausal only), age at menopause (postmenopausal only), BMI at age 18 (continuous).  Note: 3-9 MET-hours/week corresponds to an activity level comparable to approximately 1-3 hours/week of brisk walking | | | | | | | | | | | | | | |
